# Plant Growth Inhibitory Activities and Volatile Active Compounds of 53 Spices and Herbs

**DOI:** 10.3390/plants9020264

**Published:** 2020-02-18

**Authors:** Takayuki Sekine, Kwame Sarpong Appiah, Majid Azizi, Yoshiharu Fujii

**Affiliations:** 1 Miyagi Prefectural Agriculture and Horticulture Research Centre, 1, Higashi-kongouji, Kawakami, Takadate, Natori, Miyagi 981-1243, Japan; sekine-ta831@pref.miyagi.lg.jp; 2 Department of Biological Production Science, United Graduate School of Agriculture, Faculty of Agriculture, Tokyo University of Agriculture and Technology, Fuchu, Tokyo 183-8509, Japan; ksappiah90@gmail.com; 3 Department of Horticulture, College of Agriculture, Ferdowsi University of Mashhad, Mashhad 9177948974, Iran; azizi@um.ac.ir; 4 National Institute for Agro-Environmental Sciences, 3-1-3, Kan-nondai, Tsukuba, Ibaraki 305-8604, Japan

**Keywords:** allelopathy, dish pack method, herbs, lettuce growth, sandwich method, spices, total activity, volatile compounds

## Abstract

The inhibitory activities of the leachates and volatiles from 53 plant species (spices and herbs) were evaluated against lettuce (*Lactuca sativa* “Great Lakes 366”) seedling growth using the sandwich and dish pack methods, respectively. With the sandwich method, parsley (*Petroselinum sativum*) showed the strongest inhibitory effect on lettuce radicle growth (77%), followed by tarragon (*Artemisia dracunculus*) (72%). However, caraway (*Carum carvi*), dill (*Anethum graveolens*) (seed), laurel (*Laurus nobilis*), rosemary (*Rosmarinus officinalis*), and sage (*Salvia officinalis*) were the most inhibitory species (100% inhibition of lettuce radicle and hypocotyl growth inhibition at all distance wells) in the dish pack method. Cardamom (*Elettaria cardamomum*) and thyme (*Thymus vulgaris*) also showed strong inhibitory activity (100% for radicle and hypocotyl growth inhibition at all 41 and 58 mm distance wells). The headspace sampling and gas chromatography-mass spectrometry (GC-MS) analysis identified the main inhibitory active compounds as carvone in caraway and dill (seeds), 1,8-cineole in laurel and cardamom, and borneol in thyme. Both camphor and 1,8-cineole were detected in rosemary and sage, and the total activity evaluation showed that camphor was the major inhibitory compound in rosemary, although both compounds played equal roles in sage.

## 1. Introduction

A range of secondary metabolites is synthesized by plants, with the exact composition varying among species. Application of these compounds as agricultural chemicals has been well investigated, with many of the insecticides and fungicides that are used in recent years originating from natural plants [1]. The inhibitory effects of specific volatiles or essential oils of aromatic plants, including spices and herbs on plant growth have also been investigated in both field and laboratory assays [2,3,4,5]. As a result, many plant growth inhibitory substances (allelochemicals) have been identified. However, only a small number of these allelochemicals are currently being used in commercial herbicide production because their effects are not as strong or long-lasting as synthetic herbicides [1]. Nonetheless, there have been some reports on the direct use of allelopathic plants in agriculture. For example, some plants in the Brassicaceae family are used as bio-fumigation materials to reduce the incidence of soil-borne diseases, nematodes, or weeds [6,7,8,9,10].

Allelochemicals are released from plants into the environment through several routes, including volatilization from the leaf tissues, leaching of non-volatiles from the foliage by rainfall, exudation from living roots, or the decomposition of residues by soil microorganisms [11,12,13]. Several bioassay methods that correspond to each of these routes of allelochemical release have been developed to evaluate the activities of allelochemicals [3,14,15,16,17,18,19]. Among these bioassays, the sandwich method is used to evaluate the allelopathic activities of leachates by placing plant samples between two layers of agar [20,21,22]. The dish pack method is however used to evaluate the growth inhibitory activities of plant volatiles by placing samples in a six-well multi-dish to determine the relationship between the degree of growth inhibition on receptor seedlings and their distance from the donor samples. The speed of diffusion and intensity of activity of the volatiles can be estimated using this bioassay [23,24,25]. Indeed, it was through this method that Sekine et al. [26] found that cuminaldehyde was the main antifungal compound in black Zira (*Elwendia persica*). 

Therefore, in this study, we evaluated the growth inhibitory activities of the leachates and volatiles of 53 plant species (spices and herbs) using the sandwich and dish pack methods, respectively, and determined the active component(s) responsible for any such inhibitory effects.

## 2. Results and Discussion

### 2.1. Screening of Allelopathic Activity

In this study, we examined the plant growth inhibitory activities of 53 species of plants (spices and herbs) against the growth of lettuce seedlings using the sandwich and dish pack methods. The volatile compounds responsible for the activities of the most inhibitory species were also identified. Lettuce was chosen as the test plant because it germinates quickly with high uniformity and has a high sensitivity to allelochemicals [27]. In addition to this, lettuce had previously been used by many researchers to investigate plant allelopathy [18,28,29,30,31]. The inhibitory effects of the leachates and volatiles released by the spices and herbs on the radicle and hypocotyl growth of lettuce seedlings are shown in Table 1. In both the sandwich (36 species) and dish pack (23 species) methods, lettuce radicle growth was inhibited more than hypocotyl growth (Table 1). The stronger inhibition of the radicle that was observed could be the result of the radicle emerging before the hypocotyl, the nutrients stored in the seed being supplied to the hypocotyl, or differences in the actions of the allelopathic substances [27]. The allelopathic activity, in this study, was mainly discussed in terms of the lettuce radicle inhibition because the radicle is likely to be directly affected by the available leachates or volatiles, whereas hypocotyl growth is likely to be influenced by several complex factors. 

Itani et al. [27,32] found that *Oxalis corniculata*, *Rumex acetosella*, and *Begonia* spp., showed 90% to 95% inhibition when lettuce was treated with 10 mg of plant samples in the sandwich method. Using the same amount of sample, we found that none of the species tested exhibited > 90% growth inhibition with the sandwich method (Table 1). However, all of the species showed some degree of radicle growth inhibition, with parsley (*Petroselinum sativum*) showing the strongest inhibition (77%), followed by tarragon (*Artemisia dracunculus*) (72%). It has previously been reported that parsley contains myristicin and apiole both of which have shown inhibitory activities against the seedling growth of rice [33,34]. In addition, some members of the genus *Artemisia,* including tarragon, have been reported to be allelopathic [3,35,36]. However, the plant growth-inhibitory substance(s) that are specific to tarragon remain unknown. Furthermore, most of the samples inhibited hypocotyl growth, with clove (*Eugenia aromatica*) showing the strongest inhibition (72%), followed by oriental mustard (*Brassica juncea*) (69%). However, seven of the evaluated samples showed a stimulatory growth effect, with seri roots (*Oenanthe javanica*) and red shiso (*Perilla frutescens*), in particular, promoting growth by >20%. Sekine et al. [26] previously showed that 500 mg of the test sample was adequate for assessing the allelopathic activity of black Zira against the mycelial growth of *Fusarium oxysporum* using the dish pack method. Using the same amount of sample, we found that caraway (*Carum carvi*), dill seeds (*Anethum graveolens*), laurel (*Laurus nobilis*), rosemary (*Rosmarinus officinalis*), and sage (*Salvia officinalis*) caused complete inhibition of lettuce seed growth in all five wells (Table 1).

Other species including cardamom (*Elettaria cardamomum*) and thyme (*Thymus vulgaris*), also caused 100% inhibition in three of the five wells. Additionally, the pepper tree (*Schinus molle*) and mace (*Myristica fragrans*) caused strong inhibition in the two 41 mm distance wells. However, their activity sharply decreased with increasing distance (58, 82, and 92 mm) from the source well, indicating low volatility of the allelopathic compounds produced by these species. By contrast, the green and red varieties of shiso (*Perilla frutescens*) stimulated the radicle growth of lettuce by two-fold as compared with the control. It has been reported that shiso leaves contain the aromatic compound pellylaldehyde [37], and we observed that 50 μL of authentic pellylaldehyde had a similar promotional effect when tested using the dish pack method (Sekine, unpublished report). However, since this type of effect was not the focus of this study, the presence of pellylaldehyde in shiso was not further investigated in this study. The five species that showed 100% inhibition in all of the wells at a sample weight of 500 mg (excluding laurel) also showed the same level of inhibition at a reduced sample weight of 250 mg. Laurel showed less than 100% inhibition at the two furthest wells from the source well (82 and 92 mm distance wells). Furthermore, caraway, dill (seed), rosemary, and sage also showed 100% inhibition in all of the wells, even 100 mg. 

### 2.2. The GC-MS Analysis of Volatiles Constituents

The volatiles that were produced by the seven most inhibitory species (i.e., caraway, dill (seed), laurel, rosemary, sage, cardamom, and thyme) were determined through headspace sampling and GC-MS analysis. The GC-MS analysis resulted in the detection of 12 compounds (Table 2). Among these species, sage contained the most considerable number of compounds (n = 9). Among the detected compounds, limonene was present in most species (n = 6) in varying amounts, while borneol was only detected in thyme.

### 2.3. Evaluation of Allelopathic Activities of the Detected Volatiles

Evaluation of allelopathic activities of the authentic samples of the 12 detected compounds showed that borneol, camphor, carvone, and 1,8-cineole resulted in 100% growth inhibition in all of the wells. In addition, 3-carene and β-pinene exhibited high levels of inhibition when 50 μL of the compounds were used (Table 3). Furthermore, growth inhibition remained high for borneol, camphor, and carvone (>50%) when the sample volume was reduced to <50 μL or 50 mg, which equated to a vapour concentration of <1 ppm after 24 h (Table 3). The compound 1,8-cineole also showed high growth inhibition but at a higher vapour concentration than the other three compounds (10.3 ppm in the 41 mm distance well and 9.43 ppm in the 92 mm distance well). There have been several reports on the plant growth inhibitory activities of these four monoterpenoids [2,19,30,38,39,40,41,42]. Abraham et al. [2] estimated that these compounds inhibited the germination of maize (*Zea mays*) in the order of camphor > 1,8-cineole > α-pinene > limonene. 

In another study, Nishida et al. [19] reported that the root growth of *Brassica campestris* was inhibited by these compounds and β-pinene in the order of camphor > 1,8-cineole > β-pinene > α-pinene > camphene. The inhibition trends reported in both studies are similar to our findings despite the use of different types of receptor plants. Contrary to the results of this study where borneol showed higher activity than carvone, Vokou et al. [30] reported a different inhibition trend (carvone > camphor > 1,8-cineole = borneol) of some of these monoterpenoids against lettuce. This variation could be due to differences in the type of bioassay and method of vapour concentration measurement that was used. Limonene of these top four compounds was detected in six of the seven most inhibitory species, whereas both camphor and 1,8-cineole were found in rosemary and sage (Table 2). To determine which of these two compounds played a significant role in the activity of each of these species, they were further evaluated based on their specific activity (EC_50_) and total activity. Evaluation of the specific activity (i.e., biological activity per unit weight of the compound) expressed as the EC_50_ and essential for the development of pesticides, as a compound that exhibits a small EC_50_ value has a high specific activity. By contrast, the evaluation by total activity (i.e., biological activity per unit weight of the sample containing the bioactive compound) is important for biological use [43,44]. Hiradate et al. [45] isolated novel plant growth inhibitory compounds from *Spiraea thunbergii* through the concept of total activity. The EC_50_ values of authentic camphor and 1,8-cineole were 0.0633 ppm and 7.21 ppm, respectively. The total activity, based on these values and the concentration of the compounds, was calculated to be almost 10 times higher for camphor than for 1,8-cineole (23.9 and 2.58, respectively) in rosemary. Almost the same total activity for both compounds in sage (7.93 for 1,8-cineole and 7.49 for camphor) indicates that they play equal roles in the inhibitory activity of this herb.

## 3. Materials and Methods 

### 3.1. Screening of Spices and Herbs

Dried samples of 53 species of spices and herbs were tested for their potential allelopathy through leachates and volatiles (Table 1). Thirty-eight of the species were donated by YASUMA Co. Ltd. (Tokyo, Japan), 14 were cultivated in the fields of the Miyagi Prefectural Agriculture and Horticulture Research Centre (Natori, Japan) or the National Institute for Agro-Environment Science (Tsukuba, Japan), and one species was donated by the Ferdowsi University of Misshad (Iran). Each sample was dried in a hot air circulation oven at 60 °C for 4 h and, then, ground finely with a Japanese traditional grinder, “Yagen”, just before the experiment. 

### 3.2. Sandwich Method

The activity of the leachates produced by each plant sample was evaluated following the principles of the sandwich method using six-well multi-dishes (Nunc, external dimensions 128 × 86 mm, 35 mm diameter wells) [22]. Each well was filled with 10 mg of ground sample to which 5 mL of 0.5% agar (w/v) was added. The sample was, then, wholly integrated with the first layer of agar. As soon as the agar had hardened, a second agar layer (5 mL) was added and again allowed to gelatinise. Five lettuce (*Lactuca sativa* “Great Lakes 366”, Takii Seed, Japan) seeds were placed on top of the gelatinized agar in the well. Three wells of a multi-dish were used as three replications of a single species. In addition, a control multi-dish was set up in the same manner only without the addition of any samples to the wells. The multi-dishes were incubated in a dark growth chamber at 25 °C for three days. The growth rate of the lettuce seedlings relative to the control was then measured to calculate the inhibition rate (control = 100% growth). 

### 3.3. Dish Pack Method 

**Figure 1:**
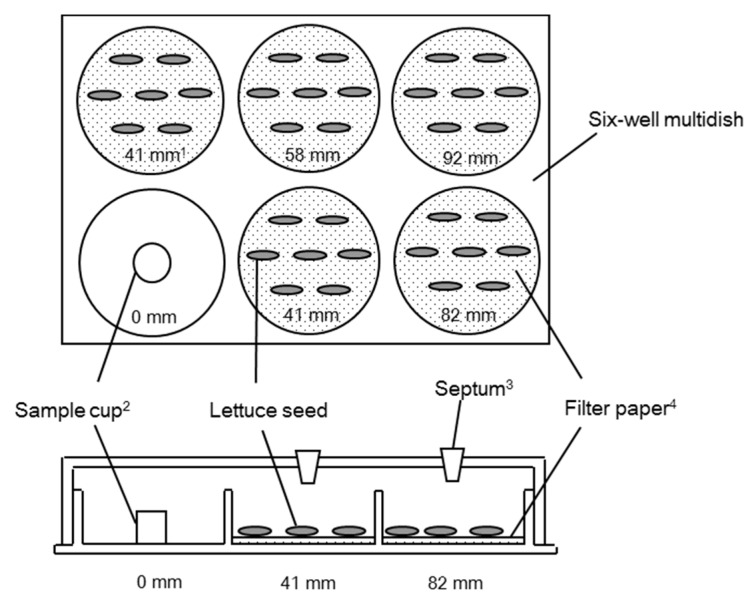
Top and side views of the multi-dish that was used for testing the plant growth inhibitory activities of volatile compounds with the dish pack method. (**^1^**) Distance from the sample or compound; (**^2^**) The source well (or cup in the case of authentic compounds); (**^3^**) Septa were attached for headspace vapour sampling, which was undertaken using a Hamilton gas-tight syringe and analyzed by gas chromatography-mass spectrometry (GC-MS) analysis; (**^4^**) Each filter paper was wetted with 0.7 mL of distilled water.

The activity of the volatiles released by each plant sample was evaluated following the dish pack method procedure [24,42] using six-well multi-dishes (Figure 1). A ground sample (500 mg) was placed in the lower-left well (denoted as the 0 mm distance or source well) of a multi-dish, and a filter paper (Advantec, No.1, 33 mm; Toyo) that had been wetted with distilled water (0.75 mL). 

Seven lettuce seeds were placed on the surface of each of the remaining wells. The multi-dish was then covered, and the sides were sealed with adhesive tape to prevent volatile losses due to volatilization. In addition, a control dish was set up in the same manner only without the addition of any sample to the source well. The multi-dishes were incubated in a dark growth chamber at 25 °C for three days. The radicle and hypocotyl lengths of the lettuce seedlings in each well were measured. The inhibition rate was, then, calculated in the same manner as for the sandwich method (see Section 3.2). There was a 1 mm headspace between the multi-dish cover and the wells to allow any volatiles produced by the sample to spread throughout the multi-dish. The levels of vapour diffusion and inhibition were estimated by the relationship between the level of seedling growth inhibition and the distance of the seedling from the source well. In this experiment, there were no replications for each species. However, those species that showed strong inhibition were assayed for a second time using a reduced amount of sample (250 mg) and this process was, then, repeated for a third time using a further reduced weight (100 mg).

### 3.4. Identification and Evaluation of Plant Growth Inhibitory Volatiles 

The volatiles that were released by the species showing the highest activities were determined using the headspace method. A finely ground sample (200 mg) of each species was put into a glass vial equipped with aluminium crimp seal cap with a polytetrafluoroethylene (PTFE)/silicone septum and was kept under laboratory conditions for 30 min. A headspace vapour sample (1 mL) was, then, taken from the vial through the septum using a Hamilton gas-tight syringe. The collected gas was then immediately injected into a QP-5050A gas spectrometer (SHIMAZU, Kyoto, Japan) using EQUITY-5 gas chromatography (GC) column (Supelco, 30 m × 0.25 mm, i.d. 0.25 μm). Each compound was identified by comparing its mass spectrum with values recorded in the NIST Mass Spectral Library and the retention time of an authentic sample on the GC. The quantity of each compound from a given plant sample was determined by comparing the peak area of the compound with that of its authentic sample. The operating conditions of the gas chromatographer-mass spectrometer were as follows: Inlet 200 °C and column oven 40 °C for 30 s and, then, programmed to increase by 8 °C/min to 160 °C, which was maintained for 5 min. The split-less injection was applied using 1.0 mL of vapour-phase sample or 2.0 μL of the liquid sample. Headspace sampling and analysis were not replicated.

To evaluate the growth inhibitory activities of authentic samples of compounds that corresponded to the detected compounds, the same procedure was applied for testing the dried samples. The only change was a cup containing 50 μL of the authentic compound (or 50 mg for solid compounds) placed in the source well instead of the dried sample (Figure 1). Authentic samples were purchased from Wako Pure Chemicals Industries, Ltd. (borneol, camphene, camphor, carvone, 1,8-cineole, limonene, α-pinene, β-pinene, and γ-terpinene), Sigma Chemical Co. Ltd. (3-carene and p-cymene), and Tokyo Chemical Industry Co. Ltd. (β-myrcene). The compounds that showed strong inhibition of lettuce seedling growth were re-assayed using a reduced amount of sample (5 μL or 5 mg until amounts of 0.5 μL or 0.05 mg were reached). Since the reduced quantities of the compounds were difficult to measure, the compounds were added to 50 μL of dimethyl sulfoxide (DMSO), which has been shown to have no effect on the germination or elongation of lettuce seeds at concentrations of 50 μL and below (Fujii, unpublished report). 

To measure the vapour concentration of the authentic compound in each well, separate multi-dishes were set out in the same manner as above but without lettuce seeds. One multi-dish corresponded to a well, and the vapour was collected from the multi-dish only once to avoid any unusual diffusion of vapour during the collection process. The vapour was collected after 24 h using a Hamilton gas-tight syringe (1.0 mL) through a septum located on top of the wells. The gas chromatography-mass spectrometry (GC-MS) analysis was undertaken using the same procedure as in Section 3.4. In addition, the biological activity of the most active commonly detected authentic compound among the tested species was evaluated based on its specific activity and total activity. Specific activity (expressed as EC_50_), is the effective concentration of the compound that induces half-maximum inhibition. In our study, the EC_50_ value was calculated using a probit analysis [46], and the total activity was calculated as:

Total activity = (1/EC_50_) × concentration in the sample (μg/g plant)

## 4. Conclusions

The leachates from parsley, followed by tarragon showed the strongest inhibitory activity against lettuce seedling growth using the sandwich method. On the other hand, the volatile constituents from caraway, dill seed, laurel, rosemary, and sage followed by cardamom and thyme showed the highest inhibition using the dish pack method. Headspace sampling and GC-MS analysis identified the main inhibitory compounds as carvone in caraway and dill seed, 1,8-cineole in laurel and cardamom, and borneol in thyme. Both camphor and 1,8-cineole were detected in rosemary and sage. Although both compounds played potentially equal roles in the inhibitory activity of sage, the total activity evaluation demonstrated that camphor was the major inhibitory compound in rosemary.

## Figures and Tables

**Table 1:** Allelopathic activities of 53 spices and herbs based on the sandwich and dish pack methods.

Donor Spice and Herb			Inhibition (%) ^3^									
			Sandwich Method ^4^		Dish Pack Method ^5^							
Common Names (Part Used) ^1^	Scientific Name	Family	R	H	Radicle				Hypocotyl			
					41	58	82	92	41	58	82	92
Onion ^2,^*	*Allium cepa*	Liliaceae	38	9	17	5	12	−1	14	21	16	13
Scallion (root)	*Allium fistuiosum*	Liliaceae	22	0	45	12	−10	−5	11	−3	−7	7
Scallion (leaf & stem)	*Allium fistuiosum*	Liliaceae	58	24	−2	9	−10	16	11	−3	−7	−5
Chinese pepper	*Zanthoxylum bungeanum*	Rutaceae	68	62	89	100	44	69	94	100	72	81
Chinpi *	*Citrus unshiu*	Rutaceae	38	13	8	4	−24	−3	3	6	−11	−23
Clove *	*Eugenia aromatica*	Myrtaceae	49	72	69	8	31	39	47	6	19	16
Allspice *	*Pimenta officinalis*	Myrtaceae	35	21	38	−27	38	12	49	36	46	18
Juniper berry *	*Juniperus communis*	Cupressaceae	11	−9	75	37	20	33	71	26	18	29
Mace *	*Myristica fragrans*	Myristicaceae	44	46	95	90	54	−4	97	92	71	51
Nutmeg *	*Myristica fragrans*	Myristicaceae	21	31	24	−2	−11	−9	58	39	18	26
Red pepper *	*Capsicum annuum*	Solanaceae	49	19	48	53	43	25	15	30	25	10
Houttuynia (root)	*Houttuynia cordata*	Saururaceae	18	−6	47	44	−1	23	44	34	−1	7
Houttuynia (leaf & stem	*Houttuynia cordata*	Saururaceae	38	−8	13	3	26	22	17	−5	40	29
Caraway *	*Carum carvi*	Umbelliferae	60	44	100	100	100	100	100	100	100	100
Dill (seed) *	*Anethum graveolens*	Umbelliferae	58	54	100	100	100	100	100	100	100	100
Dill (leaf & stem)	*Anethum graveolens*	Umbelliferae	62	37	8	22	34	−2	3	5	36	−9
Black zira	*Bunium persicum*	Umbelliferae	68	52	93	85	47	21	94	90	71	51
Coriander *	*Coriandrum sativum*	Umbelliferae	68	31	20	52	30	26	11	23	21	21
Funnel (seed) *	*Foeniculum vulgare*	Umbelliferae	41	18	48	10	7	3	72	53	34	36
Parsley *	*Petroselium sativum*	Umbelliferae	77	62	20	32	24	11	19	25	25	7
Celery (seed) *	*Apium graveolens*	Umbelliferae	46	52	21	22	22	12	18	16	13	14
Seri (root)	*Oenanthe javanica*	Umbelliferae	33	−23	20	26	9	19	10	11	1	−3
Seri (leaf and stem)	*Oenanthe javanica*	Umbelliferae	42	5	9	6	17	20	0	38	13	11
Cumin *	*Cuminum cyminum*	Umbelliferae	60	31	50	20	−37	1	79	46	8	32
Anise *	*Pinpinella anisum*	Umbelliferae	48	7	17	35	−1	−6	48	50	36	28
Ajowan *	*Trachyspermum ammi*	Umbelliferae	56	52	−60	1	−53	−12	16	29	−4	20
Cardamom *	*Elettaria cardamomum*	Zingiberaceae	29	−3	100	100	85	75	100	100	89	78
Brown cardamom *	*Elettaria cardamomum*	Zingiberaceae	14	4	58	17	17	10	63	38	3	14
Ginger *	*Zingi* *ber officinale*	Zingiberaceae	36	26	85	52	25	46	84	53	16	48
Turmeric *	*Curcuma domestica*	Zingiberaceae	19	39	−22	−23	−2	−29	16	22	10	−11
Rosemary *	*Rosemarinus officinalis*	Labiatae	59	61	100	100	100	100	100	100	100	100
Sage *	*Salvia officinalis*	Labiatae	14	20	100	100	100	100	100	100	100	100
Thyme *	*Thymus vulgaris*	Labiatae	20	33	100	100	85	64	100	100	85	63
Lavender (leaf&stem)	*Lavandula augustifolia*	Labiatae	43	62	100	100	67	58	100	100	56	58
Lavender (root)	*Lavandula augustifolia*	Labiatae	15	3	77	52	27	39	61	13	−3	7
Savory *	*Satureja hortensis*	Labiatae	21	21	29	13	24	11	27	18	25	7
Basil *	*Ocimum basilicum*	Labiatae	68	21	51	−12	−60	−10	65	27	3	27
Origan *	*Origanum vulgare*	Labiatae	11	22	−7	−37	30	14	36	19	31	4
Shiso (green)	*Perilla frutescens*	Labiatae	37	29	−64	−96	−103	−114	30	20	11	13
Shiso (red)	*Perilla frutescens*	Labiatae	49	−24	−122	−107	−104	−116	−2	−14	−20	−12
White pepper *	*Piper nigricum*	Piperaceae	67	66	85	16	56	40	82	16	63	29
Black pepper *	*Piper nigricum*	Piperaceae	60	61	15	−13	−10	−3	42	53	34	36
Laurel *	*Laurus nobilis*	Lauraceae	24	26	100	100	100	100	100	100	100	100
Cassis *	*Cinnmomum cassia*	Lauraceae	42	29	100	48	20	32	100	65	38	21
Cinnamon *	*Cinnamomum verum*	Lauraceae	42	21	65	20	10	−7	68	48	20	10
Tarragon *	*Artemisia dracunculus*	Compositae	72	40	84	33	15	0	71	65	28	16
Camomile (leaf & stem)	*Anthemis nobilis*	Compositae	59	16	40	20	24	12	17	3	11	−1
Chamomile (root)	*Anthemis nobilis*	Compositae	17	−8	50	19	−1	−9	13	11	17	−3
Chamomile (flower)	*Anthemis nobilis*	Compositae	61	39	8	25	−4	−1	5	40	−5	5
Pepper tree *	*Schinus molle*	Anacardiaceae	13	0	100	77	21	−2	100	91	32	20
Oriental mustard *	*Brassica juncea*	Brassicaceae	64	69	99	97	54	43	97	96	45	32
Yellow mustard *	*Brassica alba*	Brassicaceae	46	42	14	32	17	19	24	44	33	34
Brown mustard *	*Brassica nigra*	Brassicaceae	32	32	44	45	10	10	33	43	32	18

^1^ Where no specific part(s) are mentioned, the edible parts were used; ^2^ Spices and herbs with an asterisk (*) were donated by YASUMA Co, Ltd., while the remainder were cultivated at our research sites; ^3^ Percentage growth as compared with the control, negative values indicate the stimulation of radicle or hypocotyl growth; ^4^ Amount applied = 10 mg, all values were derived from three replicates; ^5^ Amount applied = 500 mg, values for the 41 mm distance well are the average of two wells. 41, 58, 82, and 92 indicate the distances (mm) from the source well. R; Radicle, H; Hypocotyl.

**Table 2:** Volatile compounds identified from the spices and herbs with high growth inhibitory activities.

Plant Species	Concentration (μg/g Plant) ^1^											
	α-Pinene	Camphene	β-Pinene	β-Myrcene	3-Carene	*p*-Cymene	Limonene	1,8-Cineole	γ-Terpinene	Camphor	Borneol	Carvone
Caraway	ND	ND	ND	ND	ND	ND	4.85	ND	ND	ND	ND	0.289
Dill (seed)	ND	ND	ND	ND	ND	ND	57.2	ND	0.0947	ND	ND	6.47
Cardamom	ND	ND	ND	0.0417	0.424	ND	0.288	8.21	ND	ND	ND	ND
Rosemary	9.59	2.06	ND	0.437	ND	2.66	0.543	18.6	ND	1.51	ND	ND
Sage	7.64	1.08	1.28	1.26	ND	2.39	0.419	57.2	0.136	0.474	ND	ND
Thyme	2.83	2.80	0.308	0.110	0.853	4.57	0.149	ND	ND	ND	0.968	ND
Laurel	8.76	0.724	5.78	ND	ND	8.56	ND	155	ND	ND	ND	ND
LOD	0.008	0.005	0.007	0.006	0.012	0.056	0.004	0.010	0.003	0.007	0.007	0.006

^1^ Amount of volatile compound produced by finely ground spices and herbs during 30 min of incubation. ND, not detected and <LOD, limit of detection.

**Table 3:** Plant growth inhibitory activities and vapour phase concentrations of authentic compounds.

Compound ^1^	Amount Added	Distance (mm) ^2^	Inhibition (%) ^3^		Vapour (ppm v/v) ^4^	Compound ^1^	Amount Added	Distance (mm) ^2^	Inhibition (%) ^3^		Vapour (ppm v/v) ^4^
			R	H					R	H	
α-pinene						γ-terpinene					
	50 μL	41	65.2	73.9	861		50 μL	41	80.0	90.1	101
		58	56.8	72.6				58	35.6	65.5	
		82	84.7	82.6				82	38.1	58.9	
		92	61.0	62.7	776			92	48.5	67.1	78.9
camphene							5 μL	41	−14.9	32.2	
	50 mg	41	34.9	40.0	950			58	−17.3	32.2	
		58	44.6	47.4				82	−19.9	20.3	
		82	−4.4	−25.0				92	−10.5	27.0	
		92	−54.7	−15.1	744	camphor					
β-pinene							50 mg	41	100	100	
	50 μL	41	91.6	93.3	896			58	100	100	
		58	86.5	90.5				82	100	100	
		82	77.5	81.0				92	100	100	
		92	77.5	88.6	875		5 mg	41	100	100	
	5 μL	41	39.1	16.7				58	100	100	
		58	32.7	8.8				82	100	100	
		82	36.1	14.0				92	100	100	
		92	33.6	7.0			0.5 mg	41	100	100	1.76
β-myrcene								58	100	100	
	50 μL	41	21.7	64.9	209			82	100	100	
		58	47.8	67.6				92	90.6	94.6	0.482
		82	5.7	35.1			0.1 mg	41	83.2	94.8	0.240
		92	−1.0	43.2	154			58	67.5	86.6	
3-carene								82	64.4	83.6	
	50 μL	41	91.0	89.3	580			92	49.7	70.2	
		58	88.0	91.5			0.05 mg	41	41.4	59.0	0.0472
		82	72.9	80.8				58	19.4	38.9	
		92	89.0	93.6	625			82	16.2	34.4	
	5 μL	41	38.2	17.5				92	33.0	55.3	
		58	49.7	14.0		borneol					
		82	40.4	8.8			50 mg	41	100	100	
		92	28.4	−8.8				58	100	100	
*p*-cymene								82	100	100	
	50 μL	41	52.9	70.3	103			92	100	100	
		58	19.2	51.4			5 mg	41	100	100	
		82	14.1	40.5				58	100	100	
		92	7.4	51.4	54.8			82	100	100	
								92	100	100	
limonene						(Borneol)					
	50 μL	41	3.2	32.8	183		0.5 mg	41	100	100	5.53
		58	2.5	32.8				58	100	100	
		82	−0.3	40.3				82	93.4	94.6	
		92	29.0	47.8	167			92	96.2	94.6	0.435
1,8-cineole							0.1 mg	41	81.7	78.9	0.0923
	50 μL	41	100	100				58	83.8	75.4	
		58	100	100				82	56.6	31.6	
		82	100	100				92	52.3	21.1	
		92	100	100		carvone					
	5 μL	41	100	100			50 μL	41	100	100	0.184
		58	100	100				58	100	100	
		82	100	100				82	100	100	
		92	100	100				92	100	100	
	2 μL	41	100	100			5 μL	41	64.8	84.0	0.0529
		58	100	100				58	95.9	97.3	
		82	100	100				82	82.0	89.4	
		92	100	100	34.6			92	97.2	100	
	1 μL	41	85.5	91.2	10.3		2 μL	41	28.5	70.5	
		58	90.6	91.2				58	27.9	62.0	
		82	76.1	82.5				82	33.6	71.5	
		92	82.1	87.7	9.43			92	41.4	77.2	
	0.5 μL	41	9.0	42.9	4.72						
		58	15.1	35.7							
		82	16.0	37.5							
		92	15.1	46.4							

^1^ The 12 compounds that were detected from spices and herbs with high growth inhibitory activities (i.e., caraway (*Carum carvi*), dill (*Anethum graveolens*) (seed), cardamom (*Elettaria cardamomum*), rosemary (*Rosmarinus officinalis*), sage (*Salvia officinalis*), thyme (*Thymus vulgaris*), and laurel (*Laurus nobilis*)) were used; ^2^ Distance between the source well and the other wells in a six-well multi-dish; ^3^ Percentage growth inhibition as compared with the control, 100% indicates complete inhibition, negative values indicate the promotion of radicle (R) or hypocotyl (H) growth, values for the 41 mm distance well are the average of two wells, all values were derived from a single experiment; ^4^ Vapour-phase concentrations above each well after 24 h.
